# Supplementary material for: Association between participation in the Northern Finland Birth Cohort 1966 study and use of psychiatric care services
Source: PLoS One. 2023 Mar 3;18(3):e0282714. doi: 10.1371/journal.pone.0282714 (PMC9983849; doi:10.1371/journal.pone.0282714)
Supplement: Attachment — Submitted filename: Response to Reviewers.docx [file pone.0282714.s001.docx]

Comments by the Academic Editor:

**Editor E1.** Please ensure that your manuscript meets PLOS ONE's style requirements, including those for file naming. The PLOS ONE style templates can be found at
<https://journals.plos.org/plosone/s/file?id=wjVg/PLOSOne_formatting_sample_main_body.pdf> and
<https://journals.plos.org/plosone/s/file?id=ba62/PLOSOne_formatting_sample_title_authors_affiliations.pdf>

**Response E1.** We have now revised our manuscript and restyled the manuscript and files.

**Editor E2**. In your Data Availability statement, you have not specified where the minimal data set underlying the results described in your manuscript can be found. PLOS defines a study's minimal data set as the underlying data used to reach the conclusions drawn in the manuscript and any additional data required to replicate the reported study findings in their entirety. All PLOS journals require that the minimal data set be made fully available. For more information about our data policy, please see <http://journals.plos.org/plosone/s/data-availability>.

Upon re-submitting your revised manuscript, please upload your study’s minimal underlying data set as either Supporting Information files or to a stable, public repository and include the relevant URLs, DOIs, or accession numbers within your revised cover letter. For a list of acceptable repositories, please see <http://journals.plos.org/plosone/s/data-availability#loc-recommended-repositories>. Any potentially identifying patient information must be fully anonymized.

Important: If there are ethical or legal restrictions to sharing your data publicly, please explain these restrictions in detail. Please see our guidelines for more information on what we consider unacceptable restrictions to publicly sharing data: <http://journals.plos.org/plosone/s/data-availability#loc-unacceptable-data-access-restrictions>. Note that it is not acceptable for the authors to be the sole named individuals responsible for ensuring data access.

We will update your Data Availability statement to reflect the information you provide in your cover letter.

**Response E2:** We have now modified the Data Availability Statement:

*‘Data cannot be shared publicly because the utilised data have been given for this specific study (Permission numbers: TK-53-1053-19, VRK/3285/2019-2, THL/1254/2019), and the data cannot be shared without permission from the register keepers. The same data can be applied from Findata, the Finnish Health and Social Data Permit Authority by researchers who meet the criteria for access to confidential data. Additionally, Findata, can prepare statistical tables from the registers used in this study:*

*https://findata.fi/en/permits/data-requests/’*

**Editor E3.** Please review your reference list to ensure that it is complete and correct. If you have cited papers that have been retracted, please include the rationale for doing so in the manuscript text, or remove these references and replace them with relevant current references. Any changes to the reference list should be mentioned in the rebuttal letter that accompanies your revised manuscript. If you need to cite a retracted article, indicate the article’s retracted status in the References list and also include a citation and full reference for the retraction notice.

**Response E3:** We revised the reference list (we did not identify any retracted references) and made corrections to the references: ref 7 and ref 28 (added the doi), ref 18, ref 20, ref 30 (corrected the doi) and corrected the title of the reference 32.

Comments by the Rewievers

**Reviewer #1**

**Reviewer R1.1:** The study by Dr. Kerkelä and colleagues aimed to examine whether the use of psychiatric care services differed between individuals included in the Northern Finland Birth Cohort 1966 (NFBC1966) and individuals born in 1965 and 1967. The authors found no evidence of a difference in psychiatric outcomes, suggesting that the NFBC1966 may represent the population. The results may be relevant for future research using the NFBC1966 cohort.

**Response R1.1:** Thank you for this comment.

**Reviewer R1.1:** Table 3: Suggest presenting the results in the form of N (%)

**Response R1.1**: We have now modified the table and report the results in the form of N (%).

**Reviewer R1.2.** How many comparison individuals born in 1965 and 1967 are family members (siblings or a spouse) of individuals in the NFBC1966 cohort? The visit of a family member may affect their health service use and thus dilute the impact of the cohort effect.

**Response R1.2:** Unfortunately, we do not have data of family members. We agree that this is a potential limitation of the study, and added the information in the limitations section:

*‘**Also, we did not have the information of possible family members in the study and comparison cohorts. The health care use of a family member may affect other family members.’.*

**Reviewer R1.3.** As the authors discussed in the limitation section, only a few follow-ups focused on psychiatric measurements at ages 33 and 42 years. What was the mean (SD) age of first psychiatric hospital contact? Suppose the first visit to a psychiatric specialty occurred before these follow-ups. In that case, it is less likely affected by the follow-up.

**Response R1.3**: We added the mean ages with SDs and the comparison with the Welch's t-test of the age of first visit to psychiatric specialty and first psychiatric hospitalisation to the table 3 and edited the legend of the table:

*‘Psychiatric care service use in study and comparison cohort in males and females.’*

The new analysis is mentioned in the 2.4 Statistical analysis section:
‘*Age of first visit to psychiatric specialty and age of first psychiatric hospitalization was analysed using the Welch's t-test*’

and in the 3. Results section the new comparison was mentioned:

‘*Age of first visit to psychiatric specialty or age of first psychiatric hospitalization did not differ between the study and comparison cohorts.’*

In the 4. discussion section the mean ages are mentioned:
‘*Of those 12.7% who visited psychiatric specialty, the mean ages of first visit for psychiatric specialty were between 35 and 37 years and of those 6.7% who had psychiatric hospitalization, mean ages of first psychiatric hospitalizations were between 31 and 32 years in the study cohort. The follow-ups for whole cohort including psychiatric screens were conducted at age 31 and 46, and the sub-studies focusing on psychiatric measurements at age 33 and 42.* ´

**Reviewer R1.4.** The authors studied severe psychiatric outcomes, such as psychiatric specialty visits and hospitalizations. However, it is relevant to know whether the patterns of prescription filling for psychiatric medications differ between individuals in the NFBC1966 and individuals born in 1965 or 1967.

**Response R1.4:** We agree with the reviewer that further elaborating on this point would be important. Unfortunately, we do not have data of the medications, since the registration in Finland was started in 1994 (reimbursed medication) and it was not complete before 2017 (prescribed medication). We have added the potential future research in the conclusion:

*‘The effects of participation in epidemiological follow-up studies have previously been under-examined earlier, and the results need to be replicated.* *In addition, the use of psychiatric medications might prove an important area for future research.’*

**Reviewer R2:** Thank you for letting me review this article. I suggest accept. I believe this submission will contribute to fill the gap in knowledge in whether how much questionnaires in research intervene in the health care seeking patterns.

**Response R2:** We appreciate the Reviewer’s input to review the manuscript and give this positive comment.
The manuscript has now been proofread, and the typographical and grammatical errors have been corrected as you suggested.

Additional edits:

**A1.** The manuscript has been proofread by a professional.
