# Supplementary material for: Association between participation in the Northern Finland Birth Cohort 1966 study and use of psychiatric care services
Source: PLoS One. 2023 Mar 3;18(3):e0282714. doi: 10.1371/journal.pone.0282714 (PMC9983849; doi:10.1371/journal.pone.0282714)
Supplement: Attachment — Submitted filename: Response to reviewers.docx [file pone.0282714.s002.docx]

Comments by the Academic Editor:

**Editor E1:** Please clarify if there might be any bias or interpretation between the association of participation of psychiatric patients and non-participants in the study.

**Response E1:** We are aware of selective non-participation bias in the NFBC1966. It is already known that participants of the NFBC1966 in the 31-year and 46-year studies are more often female, were less often unemployed and are from higher social class (Nordström T, Miettunen J, Auvinen J et al., 2021) and subjects with psychiatric disorders participated less actively than those without any psychiatric disorders in the 31-year study (Haapea M, Miettunen J, Läärä E, et al. 2008). But, in the present study we did not actually focus on the attrition of the NFBC1966 sample, but more in the effects of the longitudinal follow-up study, nevertheless have the subject participated in each follow-up study or not. Please see response R3.3.

References:

Nordström T, Miettunen J, Auvinen J, Ala-Mursula L, Keinänen-Kiukaanniemi S, Veijola J, et al. Cohort Profile: 46 years of follow-up of the Northern Finland Birth Cohort 1966 (NFBC1966). Int J Epidemiol. 2021; dyab109. doi:10.1093/ije/dyab109

Haapea M, Miettunen J, Isohanni MK, Veijola JM, Läärä E, Järvelin MR, et al. Non-participation in a field survey with respect to psychiatric disorders. Scand J Public Health. 2008;36. doi:10.1177/1403494808092250

**Editor E2:** Please give an an explanation or write a paragraph that could explain if there emerge any difference when the comparison cohort study is conducted  with a   reduced sample.

**Response E2:** The study group was not NFBC1966 per se, but a population born in 1966 in Northern Finland. We could not identify those who participated in the NFBC1966 and took part in follow-up studies. Nevertheless, 96.3% of the study cohort were members of the NFBC1966. Please see response R3.3

**Journal Requirements JR1:** Please review your reference list to ensure that it is complete and correct. If you have cited papers that have been retracted, please include the rationale for doing so in the manuscript text, or remove these references and replace them with relevant current references. Any changes to the reference list should be mentioned in the rebuttal letter that accompanies your revised manuscript. If you need to cite a retracted article, indicate the article’s retracted status in the References list and also include a citation and full reference for the retraction notice.

**Response JR1:** We have now revised the reference list and corrected the reference 20: Järvelin MR, Sovio U, King V, Lauren L, Xu B, McCarthy MI, Hartikainen AL, Laitinen J, Zitting P, Rantakallio P, Elliott P. Early Life Factors and Blood Pressure at Age 31 Years in the 1966 Northern Finland Birth Cohort. Hypertension. 2004;44: 838–846. doi:10.1161/01.HYP.0000148304.33869.ee

**Reviewer R3.1:** In this study, the authors examined whether use of psychiatric care services differed between individuals included in the Northern Finland Birth Cohort 1966 (NFBC1966) differed from individuals born in 1965 and 1967, their hypothesis being that study involvement may act as an intervention of sorts by motivating people to seek treatment they otherwise would not. The authors found no evidence of a difference in psychiatric outcomes, suggesting NFBC1966 is representative of the broader population. I think this could be valuable information for those using this data resource in the future. However, I have a couple of things I would like to see the authors address first...

**Response R3.1:** Thank you for the comment.

**Reviewer R3.2:** The authors' characterization of potential inks between participation and health implies that participation may cause improvement in health. This seems a little odd to me, typically associations between participation and health are interpreted through the lens of selection bias - unhealthy people are less likely to take part in studies and more likely to drop out when they do. This doesn't actually impact the value of the research, but the authors should acknowledge that where associations are detected elsewhere this is often the interpretation.

**Response R3.2** The reviewer is right, the selection bias exists in the NFBC1966 (Please see a more detailed answer for attrition and biases in Response R3.3). We were focused on the effects of being a member of the longitudinal follow-up study, more than attrition. We modified the aims of the study:

*‘**We examined the effects of being a member of the longitudinal follow-up study cohort compared to a population born in the same geographical area in Northern Finland in 1965 and 1967.’*

edited the abstract: ‘*Although the idea is not to intervene, being a member of the longitudinal follow-up study and studies conducted during follow-up may affect the target population. ‘*

introduction: ‘*Although the idea is not to intervene, being a member of the longitudinal follow-up study and studies conducted during follow-up may affect the study population in various ways.’*

also we added achapter on the effects of awareness of participation in the study in the introduction section to emphasize the aims of the study:

*‘The awareness of participation in the study can be described as the Hawthorne effect, which describes the awareness of the study participants being studied and the possible impact on their beaviour. (Jones, 1992) In the Derbyshire Smoking Study, the Hawthorne effect of repeatedly measured smoking behaviour of about 6000 adolescents was studied by questionnaire yearly from 1974 to 1978 in selected schools. The self-reported smoking habits were compared to adolescents whose smoking habits were measured first time in 1978. The findings revealed that the prevalence of smoking was lower in those schools, which had been surveyed for five years. (Murray et al., 1988). Berkhout et all. pooled the findings of 15 studies published in 2012-2022 on the Hawthorne effect in primary care or outpatient clinics or healthy subjects. In the meta-analysis, the Hawthorne effect was defined as “an aware or unconscious complex behavioural change in a study environment, related to the interaction of four biases affecting the study subjects and investigators: selection bias, commitment and congruence bias, conformity and social desirability bias, and observation and measurement bias”. The findings demonstrate that medical research is unavoidably prone to the Hawthorne effect, which restricts its external validity, beginning with the intentional or unintentional selection of the study population, resulting in blind spots in medical knowledge. (Berkhout et al., 2022)’*

Modified the discussion:

‘*No evidence of an association of being a member of a longitudinal follow-up study and studies conducted during follow-up to a use of psychiatric care services was found*.’

**Reviewer R3.3**. Analyses all compare the full 1966 cohort to the combined 1965/67 cohort. However, the 1966 cohort actually diminishes over time, as reduced numbers take part at age 1, 14, 31, and 46. Can the authors compare the reduced samples to the comparison cohort to see if differences emerge over time? This again speaks to the fact that differences between participants and non-participants are typically interpreted as selection bias, rather than evidence for positive effects of the study's participation. e.g. The authors hypothesize that study participation will increase use of psychiatric services, so the 1966 cohort will use more that the 65/67 cohort. They find no evidence for this. Would their hypothesis apply to the samples reduced by attrition? i.e. if participation increases service use then would continued participation predict service use? This would run counter to a hypothesis that selection bias would mean that service use would predict drop out. Could the authors run such analyses to test these contrasting hypotheses?

**Response R3.3.** Our study group was not NFBC1966 per se, but a population born in 1966 in Northern Finland. We could not identify those in our study cohort who really participated in the NFBC1966 and took part in follow-up studies. Nevertheless, 96.3% of our study cohort were members of the NFBC1966.

We are aware of selective non-participation bias in the NFBC1966. It is already known that participants of the NFBC1966 in the 31-year and 46-year studies are more often female, were less often unemployed and are from higher social class (Nordström T, Miettunen J, Auvinen J, et al., 2021) and subjects with psychiatric disorders participated less actively than those without any psychiatric disorders in the 31-year study (Haapea M, Miettunen J, Läärä E, et al. 2008) We did not focus in the attrition of the NFBC1966 sample, but more in the effects of being a member in a longitudinal follow-up study, nevertheless have the subject participated in each follow-up study or not. As the postal follow-up questionnaires have been sent to N=11,768 original cohort members at 1-year follow-up, N=11,768 at 14-year follow-up, N=11,543 at 31-year follow-up and N=10,331 at age of 46-years, the vast majority of members of NFBC1966 have been aware of longitudinal follow-up.

We now have discussed the attrition and source of bias in the discussion section:

*‘**The participation rate in the follow-up studies decreased from 91.2% in the 1-year study, 93.6% in the 14-year study, 77.4% in the 31-year study to 66.5% in the 46-year study. It is already known that participants of the NFBC1966 in the 31-year and 46-year studies are more often female, were less often unemployed and are from higher social class [26] and subjects with psychiatric disorders participated less actively than those without any psychiatric disorders in the 31-year study [38]. The selective non-participation bias does exist in the NFBC1966. However, as the aim of the study was to examine the effects of participation in the longitudinal follow-up study, and the postal follow-up questionnaires have been sent to N=11,870 (97.0% of original cohort members) at 1-year follow-up, N=11,768 (96.2%) at 14-year follow-up, N=11,543 (94.4%) at 31-year follow-up and N=10,331 (83.8%) at age of 46-years, the vast majority of members of NFBC1966 have been aware of longitudinal follow-up.’*

References:

Nordström T, Miettunen J, Auvinen J, Ala-Mursula L, Keinänen-Kiukaanniemi S, Veijola J, et al. Cohort Profile: 46 years of follow-up of the Northern Finland Birth Cohort 1966 (NFBC1966). Int J Epidemiol. 2021; dyab109. doi:10.1093/ije/dyab109

Haapea M, Miettunen J, Isohanni MK, Veijola JM, Läärä E, Järvelin MR, et al. Non-participation in a field survey with respect to psychiatric disorders. Scand J Public Health. 2008;36. doi:10.1177/1403494808092250
